# High Throughput Procedure for Comparative Analysis of In Vivo Cardiac Glucose or Amino Acids Use in Cardiovascular Pathologies and Pharmacological Treatments

**DOI:** 10.3390/metabo11080497

**Published:** 2021-07-30

**Authors:** Marta Tomczyk, Mariola Olkowicz, Ewa M. Slominska, Ryszard T. Smolenski

**Affiliations:** 1 Department of Biochemistry, Medical University of Gdansk, 80-210 Gdansk, Poland; eslom@gumed.edu.pl; 2 Jagiellonian Centre for Experimental Therapeutics, 30-348 Krakow, Poland; mariola.olkowicz@jcet.eu

**Keywords:** heart, mass spectrometry, catabolism

## Abstract

The heart is characterized by the prominent flexibility of its energy metabolism and is able to use diverse carbon substrates, including carbohydrates and amino acids. Cardiac substrate preference could have a major impact on the progress of cardiac pathologies. However, the majority of methods to investigate changes in substrates’ use in cardiac metabolism in vivo are complex and not suitable for high throughput testing necessary to understand and reverse these pathologies. Thus, this study aimed to develop a simple method that would allow for the analysis of cardiac metabolic substrate use. The developed methods involved the subcutaneous injection of stable ^13^C isotopomers of glucose, valine, or leucine with mass spectrometric analysis for the investigation of its entry into cardiac metabolic pathways that were deducted from ^13^C alanine and glutamate enrichments in heart extracts. The procedures were validated by confirming the known effects of treatments that modify glucose, free fatty acids, and amino acid metabolism. Furthermore, we studied changes in the energy metabolism of CD73 knock-out mice to demonstrate the potential of our methods in experimental research. The methods created allowed for fast estimation of cardiac glucose and amino acid use in mice and had the potential for high-throughput analysis of changes in pathology and after pharmacological treatments.

## 1. Introduction

Proper heart function requires a constant and substantial energy supply. To acquire the energy that is necessary to carry out its function, the heart converts chemical energy stored in cardiac substrates into the mechanical energy of the actin–myosin interaction of myofibrils. The heart cycles about 6 kg of adenosine triphosphate (ATP) every day, which is 20 to 30 times its weight [1]. However, ATP reserves are relatively low in relation to the need for high-energy phosphates necessary to maintain the metabolism and ion balance. The reserves of high-energy phosphate in the heart are very small even when considering phosphocreatine (PCr), which is present at a concentration that is twice the concentration of ATP. PCr’s role is not just as an energy reserve. This compound also participates in energy transport between the mitochondrial system and the contractile apparatus. In the mitochondrial inter-membrane space, the energy of the high-energy phosphate bond of ATP can be transferred to creatine by mitochondrial creatine kinase (CK) resulting in the formation of PCr. In the cytosol, PCr can be used to resynthesize ATP from adenosine diphosphate (ADP) by cytosolic CK. Another important feature of the energy metabolism of the heart is its dependence on oxygen metabolism. Under physiological conditions, the main cardiac source of ATP production (>90%) is an oxidative phosphorylation pathway [2]. It is well known that cardiac O_2_ consumption is 60–150 μL/min/g in the resting heart and can increase fivefold during exercise [3].

Due to increased energy demand, the heart has developed impressive metabolic flexibility for utilizing all carbon substrates, including carbohydrates, fatty acids, ketones, lactate, and branched-chain amino acids. The contribution of each of these energy substrates is strictly regulated. Interestingly, the cardiac substrate preference switches during the lifetime from primary carbohydrate (fetal and neonatal stage) to predominately fatty acids (adult) [4,5]. Furthermore, under pathological conditions, cardiac substrate preference may change adversely, which may lead to an increase in heart failure [6]. Fatty acids account for 60–80% of the total energy generated via β-oxidation. Glucose, metabolized through glycolysis, provides about 20% of the total energy. The remainder is obtained through a transformation of lactate (about 15%) and other energy substrates, i.e., pyruvate, ketones, amino acids, or acetate (about 5%) [7]. Nevertheless, some data have underlined that glucose is an optimal substrate for the ischemic or hypoxic heart due to the non-oxidative generation of ATP via glycolysis and better oxygen efficiency of oxidative ATP formation. Furthermore, some data have underlined increased glucose use for cellular structure formation in pressure overload-induced cardiac hypertrophy [8]. The most common heart failure therapies are aimed increasing the heart’s oxidative capacity by increasing glucose metabolism or reducing cardiac fatty acid utilization. It has to be mentioned that in some clinical conditions such as sepsis, a fuel shift from fatty acids to glucose may be detrimental rather than protective [9]. Recently, the role of ketone bodies or branched-chain amino acids (BCAA) in the restoration of cardiac metabolism has been underlined [10,11,12,13]. On the other hand, increased serum ketone body levels were independently associated with cardiovascular events and all-cause death in patients receiving hemodialysis [14]. Therefore, tracking the cardiac energy metabolism in diverse scenarios of heart failure events might provide new insight into the mechanism of diseases and help to optimize the therapy.

The use of energetic substrates in cardiac metabolism could be examined coordinately by the accumulation of their intermediates in the heart by metabolomic profiling; nevertheless, it does not assess the activity of metabolic pathways per se. The most common experimental methods used for metabolic investigation of cardiac substrates are based on ex vivo rat or mouse heart perfusion with radiolabeled substrates or stable isotopes [15,16]. Those methods have many advantages, such as the ability to precisely manipulate perfusate composition and cardiac loading, monitor contractile function, and introduce other experimental interventions such as ischemia. However, they are also linked with some disadvantages: the use of a saline buffer in perfusion, isolation from physiological in vivo conditions intrinsic and extrinsic to the heart, and potential changes resulting from heart excision itself. Therefore, in vivo analyses may provide parallel information to that obtained in isolated hearts. Thus, our study aims to determine the in vivo glycolytic and oxidative metabolism of labeled ^13^C glucose as well as a breakdown of labeled ^13^C leucine and ^13^C valine in mouse hearts. We also tested our methods under pharmacological therapies including glucose, free fatty acids, branched-chain amino acid metabolism activators, and inhibitors, and finally analyzed them in one of the cardiovascular disease mouse models.

## 2. Results

### 2.1. Cardiac Substrate Preference Methods Conditions

#### 2.1.1. Comparison of ^13^C Glucose Intravenous Infusion and Subcutaneous Injection

**Figure 1:**
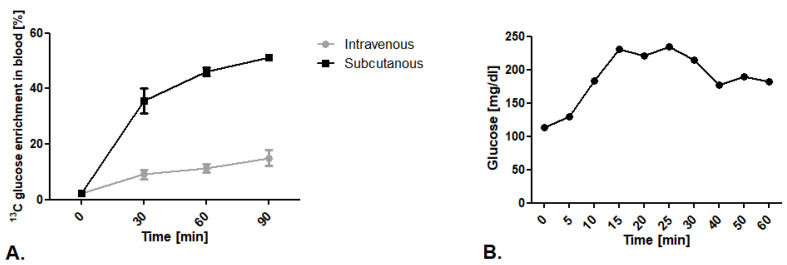
Establishment of ^13^C glucose administration route. (**A**) ^13^C glucose enrichment in blood after intravenous infusion in 0.2 mg/g body weight dose/min and subcutaneous injection of 1,6-^13^C_2_ glucose in a 1.8 mg/g body weight dose. (**B**) Glucose concentration in mouse blood after ketamine/xylazine anesthesia mixture (50 mg/kg + 5mg/kg). Results presented as mean ± SEM, *n* = 5.

The first step of our research was to establish the route of glucose isotopomer administration. We tested well-described intravenous ^13^C glucose infusion in 0.2 mg/g body weight dose/min as well as an innovative subcutaneous injection in a 1.8 mg/g body weight dose. We noted higher levels of ^13^C glucose enrichment in mouse blood after subcutaneous injection in comparison to the intravenous infusion (Figure 1A). To explain this phenomenon, we measured blood glucose levels during vein infusion.We noted that mice kept in a state of prolonged anesthesia within a ketamine and xylazine mixture experience increased blood glucose levels that translate into higher ^12^C glucose levels, and thus less measured ^13^C glucose enrichment in the blood (Figure 1B).

#### 2.1.2. Optimization of Methods Conditions

**Figure 2:**
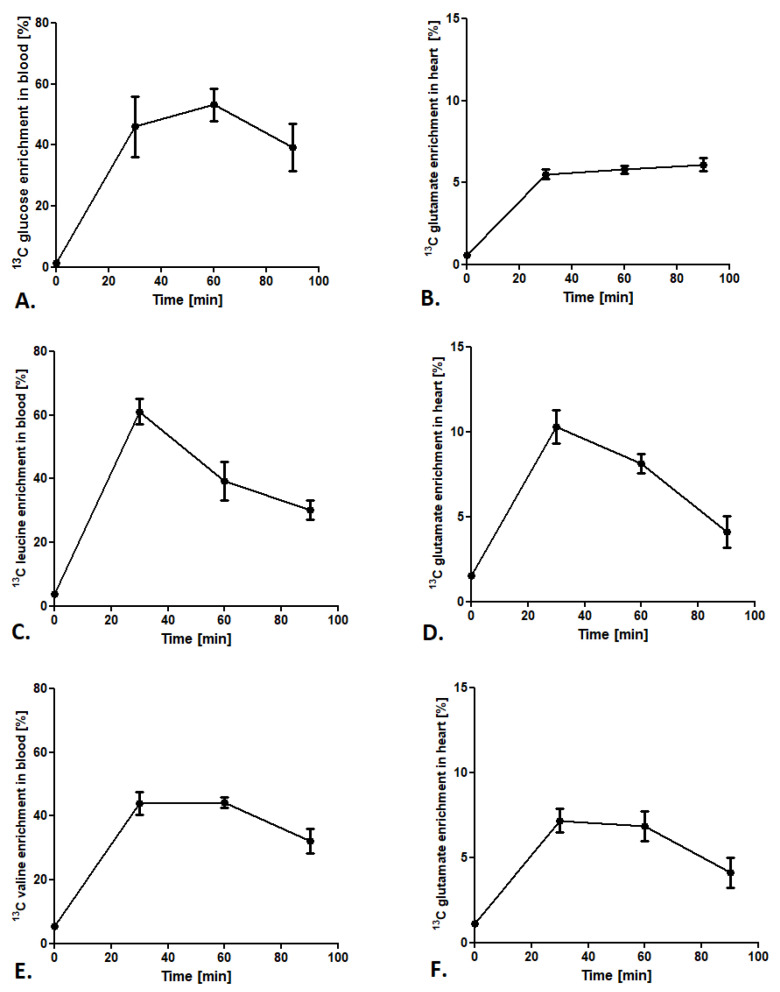
Cardiac substrate preference method conditions. (**A**) ^13^C glucose enrichment in blood after subcutaneous injection of 1,6-^13^C glucose in a 1.8 mg/g body weight dose. (**B**) ^13^C glutamate enrichment in heart tissue after a subcutaneous injection of 1,6-^13^C glucose in a 1.8 mg/g body weight dose. (**C**) ^13^C leucine enrichment in blood after a subcutaneous injection of leucine-3-^13^C in a 0.3 mg/g body weight dose. (**D**) ^13^C glutamate enrichment in heart tissue after a subcutaneous injection of leucine-3-^13^C in a 0.3 mg/g body weight dose. (**E**) ^13^C valine enrichment in blood after a subcutaneous injection of valine-1,2,3,4,5-^13^C_5_ in a 0.7 mg/g body weight dose. (**F**) ^13^C glutamate enrichment in heart tissue after a subcutaneous injection of valine-1,2,3,4,5-^13^C_5_ in a 0.7 mg/g body weight dose. Results presented as mean ± SEM, *n* = 5.

To provide a stable state of labeled glucose and BCAA during analysis, the ^13^C isotopomers enrichment in the blood and the ^13^C glutamate enrichment in the heart were tested. After administration of glucose-1,6-^13^C_2_ in a 1.8 mg/g body weight dose, ^13^C glucose enrichment (glucose-1,6-^13^C_2_/^12^C glucose ratio) in the blood after 60 min of injection achieved the maximum value of 50% (Figure 2A). Furthermore, analysis of ^13^C glutamate enrichment (4-^13^C-glutamate/^12^C glutamate ratio) in the heart revealed a constant increase from 30 min after glucose-1,6-^13^C_2_ injection and was maintained in a stable 5–6% level until 90 min (Figure 2B). In the case of leucine-3-^13^C administration, ^13^C leucine enrichment in the blood (leucine-3-^13^C/^12^C leucine ratio) increased after 30 min of injection to almost 60% and fell at a constant rate until 90 min (to 30% of enrichment) (Figure 2C). Interestingly, ^13^C glutamate enrichment (4-^13^C glutamate/^12^C glutamate ratio) in mouse heart achieved the maximum 10% only 30 min after ^13^C leucine injection and dropped to 8% at 60 min and 4% at 90 min (Figure 2D). A similar tendency in BCAA enrichment in the blood was observed in ^13^C valine enrichment. ^13^C valine enrichment (valine-1,2,3,4,5-^13^C_5_/^12^C valine ratio) grew to the maximum 40% in 30 min and then slowly fell to 30% at 90 min after injection (Figure 2E). Moreover, ^13^C glutamate enrichment (1,2,3-^13^C_3_ glutamate/^12^C glutamate ratio) in the heart after ^13^C valine administration revealed that the maximum enrichment was accomplished at 30 min after injection (about 7%), whereas at 60 and 90 min it slowly fell to 4% (Figure 2F).

### 2.2. Cardiac Substrate Preference Analysis under Pharmacotherapy Conditions

**Figure 3:**
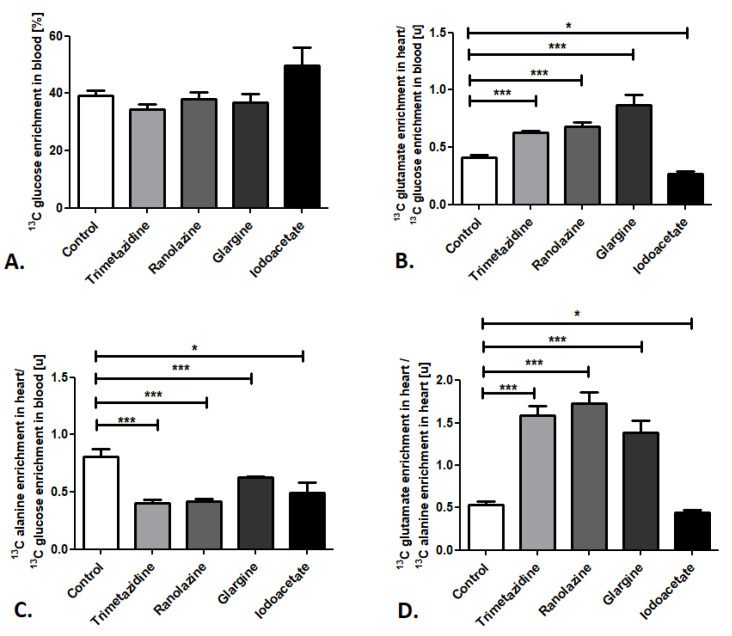
Pharmacological modulation of cardiac glucose use in mice. (**A**) ^13^C glucose enrichment in mouse blood. (**B**) ^13^C glutamate enrichment in heart/^13^C glucose enrichment in mouse blood ratio. (**C**) ^13^C alanine enrichment in heart/^13^C glucose enrichment in mouse blood ratio. (**D**) ^13^C glutamate/^13^C alanine ratio in mouse heart after pharmacological treatment. Results presented as mean ± SEM, *n* = 5, * *p* < 0.05, *** *p* < 0.001.

Next, we determined the sensitivity of the established method for changes in cardiac substrate utilization using common pharmacological compounds that activate or inhibit the glucose and free fatty acid metabolic pathways—trimetazidine, ranolazine, glargine, and iodoacetate. Importantly, administration of all of the investigated compounds did not affect ^13^C glucose enrichment in the mouse blood when it was compared to the control (Figure 3A). Nevertheless, significant alterations were observed in ^13^C glutamate enrichment in mouse heart/^13^C glucose enrichment in the blood ratios (Figure 3B). Increases in this ratio were noted after administration of trimetazidine, ranolazine (inhibitors of free fatty acid metabolism), and glargine (analog of human insulin that increases peripheral glucose disposal), whereas iodoacetate (glycolysis inhibitor) treatment resulted in reduced ^13^C glutamate enrichment in mouse heart/^13^C glucose enrichment in the blood ratio (Figure 3B). Moreover, administration of all of the investigated compounds (trimetazidine, ranolazine, glargine, and iodoacetate) resulted in diminished ^13^C alanine enrichment in mouse heart/^13^C glucose enrichment in the blood ratio in comparison to the control (Figure 3C). Summarizing, pharmacological inhibition of free fatty acid (FFA) metabolism (using trimetazidine and ranolazine) and activation of glucose use (glargine) caused the elevation in the ^13^C glutamate/^13^C alanine ratio in mouse heart, whereas glucose metabolism inhibition (iodoacetate) resulted in the reduction of ^13^C glutamate/^13^C alanine ratio in mouse heart (Figure 3D).

**Figure 4:**
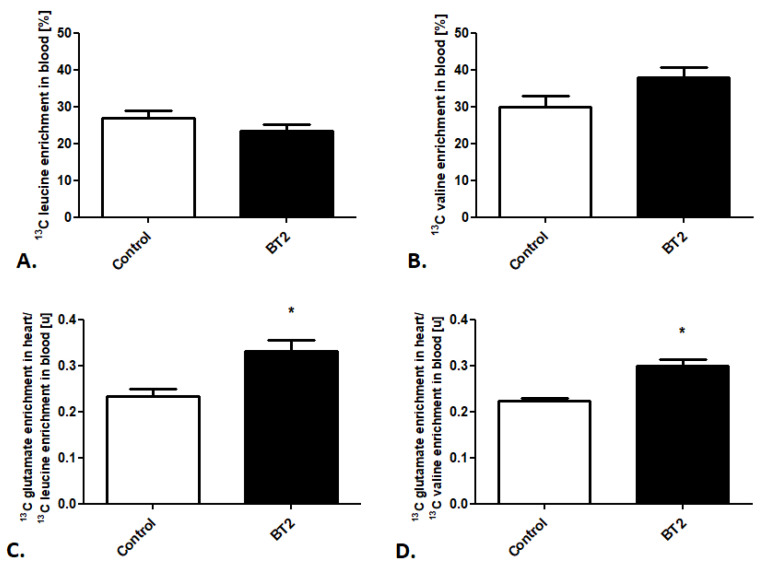
Pharmacological modulation of cardiac BCAA use in mice. (**A**) ^13^C leucine enrichment in mouse blood. (**B**) ^13^C valine enrichment in mouse blood. (**C**) ^13^C glutamate enrichment in heart/^13^C leucine enrichment in mouse blood ratio. (**D**) ^13^C glutamate enrichment in heart/^13^C valine enrichment in mouse blood after pharmacological treatment. Results presented as mean ± SEM, *n* = 5, * *p* < 0.05.

Afterward, using the created method, we also tracked cardiac BCAA metabolism after BT2 treatment. Besides stable ^13^C leucine and ^13^C valine enrichment in the mouse blood in the control and treated groups (Figure 4A,B), we noted increased ^13^C glutamate enrichment in heart/^13^C leucine enrichment in the mouse blood as well as ^13^C glutamate enrichment in heart/^13^C valine enrichment in the mouse blood ratios (Figure 4C,D).

### 2.3. Cardiac Substrate Preference Analysis in Mouse Model of Cardiovascular Disease

**Figure 5:**
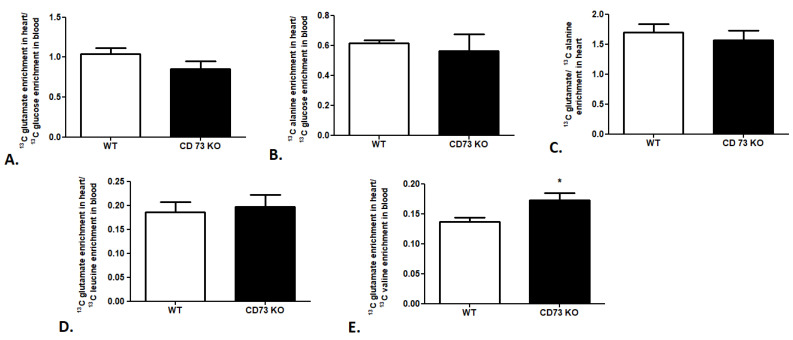
Cardiac glucose and BCAA use in CD73 KO mice. (**A**) ^13^C glutamate enrichment in heart/^13^C glucose enrichment in mouse blood ratio. (**B**) ^13^C alanine enrichment in heart/^13^C glucose enrichment in mouse blood ratio. (**C**) ^13^C glutamate/^13^C alanine ratio in mouse heart. (**D**) ^13^C glutamate enrichment in heart/^13^C leucine enrichment in mouse blood ratio. (**E**) ^13^C glutamate enrichment in heart/^13^C valine enrichment in mouse blood ratio. Results presented as mean ± SEM, *n* = 5, * *p* < 0.05.

In the last step of our research, we attempted to evaluate the cardiac substrate preference in a CD73-deficient (CD73 KO) mouse model. We observed no changes in ^13^C glutamate enrichment in heart/^13^C glucose enrichment in the mouse blood ratio, ^13^C alanine enrichment in heart/^13^C glucose enrichment in the mouse blood ratio, or ^13^C glutamate/^13^C alanine ratio in CD 73 KO mouse heart relative to wild-type mice (WT), which indicates similar glucose use in cardiac metabolism in both mouse strains (Figure 5A–C). The same tendency was observed in the analysis of leucine use in cardiac metabolism. We noted no changes in ^13^C glutamate enrichment in heart/^13^C leucine enrichment in the CD73 KO mouse blood ratio in comparison to WT (Figure 5D). However, increased ^13^C glutamate enrichment in heart/^13^C valine enrichment in the CD73 KO mouse blood ratio relative to WT was highlighted (Figure 5E).

## 3. Discussion

**Figure 6:**
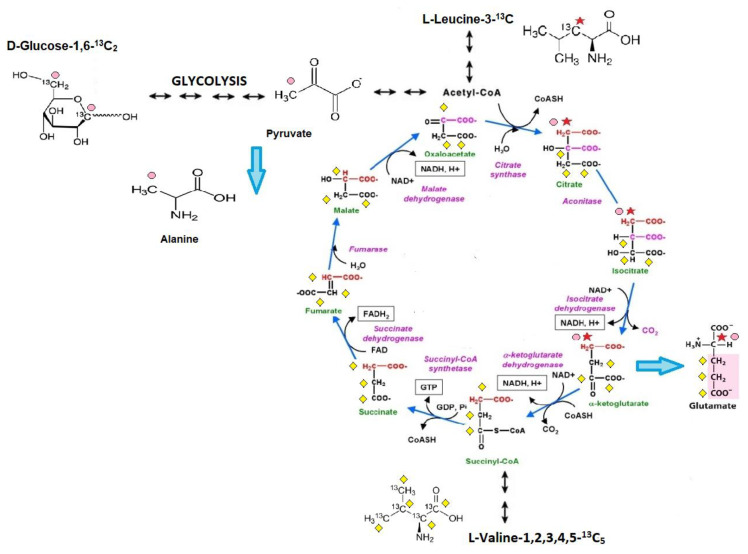
Scheme of cardiac substrate preference method. **1**. Glucose-1,6-^13^C^2^ is converted via glycolysis into 2 molecules of 3-^13^C pyruvate, which is in isotopic equilibrium with 3-^13^C alanine (pink dots reflect a ^13^C enrichment related to glucose-1,6-^13^C^2^ catabolism). Afterward, 3-^13^C pyruvate undergoes decarboxylation to 2-^13^C acetyl-CoA (catalyzed by a pyruvate dehydrogenase complex) and enters the Krebs cycle. In the first Krebs cycle turnover, the amount of 4-^13^C α-ketoglutarate, one of the products of the sequences of Krebs cycle reactions, is in balance with the intracellular 4-^13^C glutamate pool. **2.** Leucine-3-^13^C metabolism is initially catalyzed by the branched-chain amino acid aminotransferase enzyme, producing α-ketoisocaproate (α-KIC). α-KIC could be metabolized by branched-chain α-ketoacid dehydrogenase, which converts it to isovaleryl-CoA or 4-hydroxyphenylpyruvate dioxygenase (KIC dioxygenase), which converts α-KIC to β-hydroxy β-methyl butyric acid (HMB). Isovaleryl-CoA is subsequently metabolized by isovaleryl-CoA dehydrogenase and converted to methylcrotonyl-CoA, which is used in the synthesis of acetyl-CoA and other compounds, whereas the metabolism of HMB is catalyzed by an uncharacterized enzyme that converts it to β-hydroxy β-methylbutyryl-CoA (HMB-CoA). HMB-CoA is metabolized by either enoyl-CoA hydratase or another uncharacterized enzyme, producing β-methylcrotonyl-CoA (MC-CoA) or hydroxymethylglutaryl-CoA (HMG-CoA), respectively. HMG-CoA is then cleaved by HMG-CoA lyase into acetoacetate and 2-^13^C acetyl, and in this form enters the TCA cycle (red star reflects a ^13^C enrichment related to leucine-3-^13^C catabolism). In the first TCA cycle turnover, the intracellular 4-^13^C glutamate pool is monitored during the analysis (it reflects the amount of 4-^13^C α-ketoglutarate). **3.** Catabolism of valine-1,2,3,4,5-^13^C_5_ starts with the removal of the amino group by transamination, giving α -ketoisovalerate, an α -keto acid that is converted into isobutyryl-CoA through oxidative decarboxylation by the branched-chain α-ketoacid dehydrogenase complex. This is further oxidized and rearranged into 1,2,3-^13^C_3_-succinyl-CoA, which enters the citric acid cycle (yellow diamond reflects a ^13^C enrichment related to valine-1,2,3,4,5-^13^C_5_ catabolism). 1,2,3-^13^C_3_-succinyl-CoA is converted to 1,2,3-^13^C_3_ α-ketoglutarate, which is, as previously described, in the balance with the intracellular 1,2,3-^13^C_3_ glutamate pool.

This study presents an in vivo method to analyze cardiac substrate metabolic preference that relies on the subcutaneous injection of ^13^C glucose or ^13^C BCAA (^13^C leucine or ^13^C valine) and liquid chromatography with mass spectrometry (LC/MS) analysis for 3-^13^C alanine (only for ^13^C glucose metabolism tracing) and 4-^13^C glutamate (after ^13^C glucose and ^13^C leucine injection) or 1,2,3-^13^C_3_ glutamate enrichment (only for ^13^C valine metabolism tracing). Metabolite tracking (4-^13^C glutamate and 3-^13^C alanine) after ^13^C glucose administration was previously extensively studied in the early 1990s, especially within NMR analysis [17]. Theoretical assumptions, supported by experimental studies, indicate that after ^13^C glucose administration the heart accumulates 3-^13^C pyruvate in proportion to the fraction of glycolytic substrate supplied by exogenous glucose relative to alternative unlabeled substrate sources (e.g., endogenous glycogen) and 4-^13^C α-ketoglutarate in proportion to the fraction of tricarboxylic acid (TCA) cycle carbon flux supported by flux through pyruvate dehydrogenase (PDH), relative to other acetyl-CoA sources (e.g., free fatty acids (FFA)). It has to be mentioned that 3-^13^C pyruvate, as well as 4-^13^C α-ketoglutarate, were present in small quantities in the heart but occurs in isotopic equilibrium with tracked 3-^13^C alanine and 4-^13^C glutamate [18]. This is supported by the fact that Weiss et al. observed the isotopic steady state in the glutamate carbon-4 position as well 3-^13^C alanine formation in rat hearts after ^13^ C glucose administration [15,19,20]. In the case of ^13^C leucine metabolism tracking, there is a study that indicated the formation of both [1,2-^13^C_2_] and [2-^13^C]acetyl-CoA from U-^13^C leucine and a suggested estimation of α-ketoglutarate formation from C4 doublet of [4,5-^13^C_2_]glutamate [21]. Nevertheless, in our study 3-^13^C leucine was used; therefore, formation only of 4-^13^C glutamate is achievable. Based on earlier studies of ^13^C valine metabolism, 1,2,3-^13^C_3_-succinyl-CoA derived from valine-1,2,3,4,5-^13^C_5_ catabolism entered the citric acid cycle and resulted in 1,2,3-^13^C_3_ α-ketoglutarate formation, which is, as previously described, in the balance with the intracellular 1,2,3-^13^C_3_ glutamate pool [22]. Moreover, in detail, in our methods, before and at the endpoint of the analysis, mouse blood was collected and analyzed for ^13^C glucose or ^13^C BCAA enrichment. Then, the final ^13^C alanine or ^13^C glutamate enrichments were recounted for ^13^C substrates (glucose or BCAA) enrichment in the mouse blood. Thus, the measurement of myocardial 3-^13^C alanine/^12^C alanine and 4-^13^C glutamate/^12^C glutamate in steady-state ^13^C glucose enrichment in the blood allows for the estimation of the contribution of circulating glucose to myocardial glycolytic and oxidative flux, whereas 4-^13^C glutamate/^12^C glutamate in steady-state ^13^C leucine enrichment in the blood and 1,2,3-^13^C_3_ glutamate/^12^C glutamate in steady-state ^13^C valine enrichment in the blood allows for the assessment of myocardial leucine or valine usage relative to competing for metabolic substrates (Figure 6).

It is well known that mass spectrometry is a valuable approach for measuring the concentrations of metabolic intermediates [23]. The proportion of each intermediate (which is a few units heavier as a result of ^13^C labeling) is directly detectable, and thus, differences in the ratios of ^13^C-labeled metabolic substrate oxidation are detected in the labeling patterns. Stable isotope labeling is commonly used for the study of metabolic fluxes, mainly to investigate the proportional contribution of different substrates to the Krebs cycle under varying conditions and across different tissues [24,25,26]. Nevertheless, analysis of cardiac substrate preference requires specific method conditions. During the experiment, all investigated intracellular metabolic processes must be tracked. It is also important to, besides administer the exogenous substance, keep the metabolic balance. We established that after intravenous infusion of ^13^C glucose, mice suffer from sudden hyperglycemia due to prolonged ketamine/xylazine anesthesia. Moreover, there are data indicating that intraperitoneal glucose injection is also related to a significantly greater increase in blood glucose levels than oral administration [27]. That phenomenon falls gradually 30 min after injection. Nevertheless, the currently used methods are based on vein infusion or intraperitoneal injection of isotopomers and heart freeze clamping within the first 30 min after glucose bolus when its concentration in the blood may rise to unnatural levels [28,29]. Therefore, it seems important to us to create an independent method of administration of ^13^C isotopomers in mice not based on vein infusion or intraperitoneal injection. Thus, the created method allows the metabolism of ^13^C glucose and ^13^C BCAA to be tracked after simple subcutaneous injection that is able to reach stable and 40% isotopomer enrichment without a hyperglycemic event. Besides metabolic balance control, the duration of the experiment also has to be strictly established for correct evaluation of data. After the isotopomer injection, the isotopic enrichment of all intracellular metabolites that go into the transition phase, eventually reaching a steady state, should be assessed. It is also necessary to evaluate the maximum isotopomer distribution [30,31,32]. To accomplish the aforementioned requirements, the dynamics of isotopic ^13^C glucose and ^13^C BCAA enrichment in mouse blood, as well as ^13^C glutamate enrichment in the heart extracts, were determined. Based on the obtained data, we selected 90 min after ^13^C glucose injection as the time for artificial mouse ventilation and heart freeze-camping. In the case of the measurement of ^13^C BCAA use in cardiac metabolism, we chose one hour after ^13^C BCAA administration as the end point of the analysis. During this time, the smallest fluctuations in both investigated parameters, ^13^C isotopomer enrichment in the blood and ^13^C glutamate enrichment in the heart, were observed.

Years of experiments and clinical trials with pharmaceutical compounds have highlighted that modulation of the cardiac metabolism might be an interesting target for cardiovascular disease treatment. In our study, to validate the created methods, we used three well-known drugs—trimetazidine, ranolazine, and glargine. Trimetazidine is a piperazine-derivate drug that inhibits oxidative phosphorylation by shifting energy production from fatty acid to glucose oxidation, which was also shown with our method. It is caused by a selective block of activity of the last beta-oxidation enzyme, long-chain 3-ketoacyl coenzyme A thiolase (3-KAT) [33]. It is well known that trimetazidine preserves phosphocreatine and ATP intracellular levels in the failing heart [34]. Another piperazine-derivate drug similar to trimetazidine is ranolazine. Ranolazine modulates the late sodium current, thereby reducing the accumulation of intracellular Ca^2+^ [35]. Furthermore, ranolazine also results in the enhancement of glucose oxidation under a variety of conditions, including ischemia and reperfusion [36]. This is consistent with our data, which highlighted increased glucose use in glycolysis, use of acetyl-CoA in the Krebs cycle, and overall glucose use in cardiac metabolism after ranolazine treatment. The next pharmaceutic, glargine, is a first-generation long-acting basal insulin analog that regulates glucose metabolism. Insulin and its analogs lower blood glucose levels by stimulating peripheral glucose uptake, especially by skeletal muscle and fat, and by inhibiting hepatic glucose production [37]. We established that glargine treatment also results in enhanced cardiac glucose use. Interestingly, there is a study suggesting that insulin glargine might be associated with a lower risk of acute myocardial infarction, compared to the other long-/intermediate-acting insulin use [38]. It might be caused by its beneficial change in cardiac substrate metabolism. The other two compounds used in our research, iodoacetate, and BT2, are not commonly used in human research. Iodoacetate is a well-known irreversible inhibitor of the glycolytic enzyme glyceraldehyde-3-phosphate dehydrogenase (GAPDH). We noted that the created method was also able to track the reduction of glycolysis and cardiac glucose use after iodoacetate treatment. The last compound used, BT2, is an allosteric inhibitor of the branched-chain α-ketoacid dehydrogenase (BCKDC) kinase. BT2 binding to BDK results in the dissociation of BDK from BCKDC, accompanied by accelerated degradation of the released kinase in vivo. Inhibiting BCKDK activity by BT2 can effectively activate BCKDH in various tissues, leading to enhanced BCAA oxidation [39]. Recently, Uddin et al. underlined that BCAA oxidation was significantly increased in mouse heart after ex vivo perfusion with BT2 relative to the vehicle [13]. This is consistent with our data, which indicated enhanced leucine and valine use in cardiac metabolism after BT2 treatment.

Another important tool in cardiac substrate metabolism research is animal experimental models. Interestingly, the first animal model used in the investigation of cardiac substrate preference with ^13^C isotopomers was rats [40]. Nevertheless, it has to be mentioned that the use of the same methodology in mice generates many analytical problems. As an example of the application of the method in mouse research models, we used CD73-deficient mice. CD73 (ecto-5′-nucleotidase) is one of the extracellular enzymes that is involved in nucleotide catabolism and hydrolyzes adenosine-5′-monophosphate (AMP) with the release of adenosine. CD73 is found in a variety of tissues, including the heart [41]. CD73-derived adenosine is involved in cardioprotective mechanisms and vasodilatation. CD73 knock-out results in a decrease in basal coronary flow, increased fibrosis, and greater cardiomyocyte hypertrophy after a transverse aortic constriction procedure [42,43]. Moreover, our earlier studies indicated that the deletion of CD73 leads to aortic valve and endothelial dysfunction [44,45]. It is well known that deterioration in heart and vascular function may result in changes in cardiac metabolism. Thus, we chose CD73 KO mice to investigate ^13^C glucose as well as ^13^C BCAA use in cardiac metabolism. Aside from no changes in glucose oxidation and its use in the Krebs cycle, we noted an increased use of one of the investigated BCAAs—valine in CD73 KO mouse cardiac metabolism. This is in line with our data that indicated a diminished cardiac level of valine with no changes in concentration of this AA in CD73 KO mouse serum [45]. Interestingly, recently, using the created methods, we investigated the cardiac substrate metabolism in the other mouse models of cardiovascular disease—the apolipoprotein E and LDL receptor double knock-out mouse (ApoE/LDLR KO), which is one of the common models of atherosclerosis, as well as the Tgαq*44 mouse, a mouse model of cardiac hypertrophy [46,47]. In our earlier research, we also tracked glucose usage not only in mouse hearts but also in skeletal muscle [48]. Moreover, we established that cardiac substrate preference disruption might be an important factor for developing heart failure in non-cardiovascular disorders as well, like Huntington’s disease [49]. This suggests that investigation of cardiac glucose, as well as BCAA metabolism via our method, could be a useful tool for the examination of cardiac substrate preference changes not only after pharmacological treatment but also in mouse models of diseases.

## 4. Materials and Methods

### 4.1. Animals

All experiments were conducted following the Guide for the Care and Use of the Laboratory Animals published by the European Parliament, Directive 2010/63/EU, and were approved by the local bioethical committee for the Medical University of Gdansk. Animals were maintained on a 12:12 h light–dark cycle at 25 °C, 30–40% humidity, and were provided with free access to water and a standard chow diet. Six-month-old C57BL/6J and CD73 knock-out mice were used in the study.

#### 4.1.1. Administration of Stable ^13^C Glucose or ^13^C Branched-Chain Amino Acid Isotopomers

d-glucose-1,6-^13^C_2_ was administrated by intravenous infusion in 0.2 mg/g body weight dose/min under ketamine/xylazine anesthesia (50 mg/kg + 5 mg/kg) only during administration route testing. Other experiments were conducted after subcutaneous injection of a 1.8 mg/g body weight dose into pinched skin. Blood samples were collected from the tail vein before and after 30, 60, and 90 min of ^13^C_2_ glucose administration. Next, after animal anesthesia, hearts were rapidly excised (after 30, 60, 90 min), and freeze clamped after animal intubation and under artificial ventilation.

l-leucine-3-^13^C and L-valine-1,2,3,4,5-^13^C_5_ were injected subcutaneously in 0.3 mg/g (^13^C leucine) and 0.7 mg/g (^13^C valine) bodyweight doses. Blood samples were collected from the tail vein for estimation of ^13^C leucine or ^13^C valine enrichment before and after 30, 60, and 90 min of injection. The mice were anesthetized with a ketamine/xylazine mixture and the hearts were freeze clamped (also after 30, 60, and 90 min of ^13^C leucine or ^13^C valine administration).

#### 4.1.2. Administration of Glucose, Fatty Acids, and Branched-Chain Amino Acid Metabolic Inhibitors

C57BL/6J mice were randomly assigned to one of five experimental groups. The first three groups tested the effect of fatty acid metabolism inhibitors (trimetazidine, ranolazine) or the activator of glucose use (glargine) on the cardiac substrate preference method. Trimetazidine and ranolazine were administered intraperitoneally in a 30 mg/kg body weight dose for 7 days. Glargine was administrated at 0.15 mU/g intraperitoneally for 7 days. Another group tested the effect of glucose metabolism inhibition by iodoacetate, which was administered intraperitoneally for 7 days in a 30 mg/kg body weight dose. The control group was injected with 0.9% NaCl. The last one, using the 3,6-dichlorobenzo[b]thiophene-2-carboxylic acid, known as BT2, tested the effect of BCAA metabolic pathway modulation. BT2 was dissolved in dimethyl sulfoxide (DMSO) and injected intraperitoneally (final DMSO concentration of 0.5%) for 7 days in a 30mg/kg body weight dose. The control group for BT2 treatment was injected with 0.5% DMSO.

### 4.2. Blood Extraction

Blood extraction was performed using ice-cooled acetone in a 1:3 ratio (analysis of ^13^C glucose enrichment) or 1.3 M perchloric acid in a 1:1 ratio (analysis of ^13^C leucine and ^13^C valine enrichment). Next, samples were placed in ice for 15 min and centrifuged at 4 °C, 14,000 RPM/min for 10 min. In the case of cold acetone extraction, this was followed by drying in a vacuum concentrator (JW Electronic, Warsaw, Poland) and sediments were dissolved in high-purity water (Nanopure—ultrapure water system, Barnstead, Thermo, USA) and analyzed with LC/MS. Supernatants collected after centrifugation of the blood samples extracted with 1.3 M perchloric acid were neutralized with 3M K_3_PO_4_, placed in ice for 15 min, and centrifuged in the parameters mentioned above. Afterward, the supernatants were analyzed by the LC/MS method.

### 4.3. Heart Extraction

Freeze-clamped hearts were placed for 24 h in a freeze dryer (Modulyo, Thermo Electron Corporation, Waltham, MA, USA), at −55 °C. Freeze-dried fragments of hearts were extracted with 0.4 M perchloric acid in a 1:10 ratio, followed by neutralization with 2 M KOH. Supernatants obtained from centrifugation at 4 °C, 14,000 RPM/min for 10 min were analyzed by LC/MS.

### 4.4. LC/MS Methods

**Figure A1:**
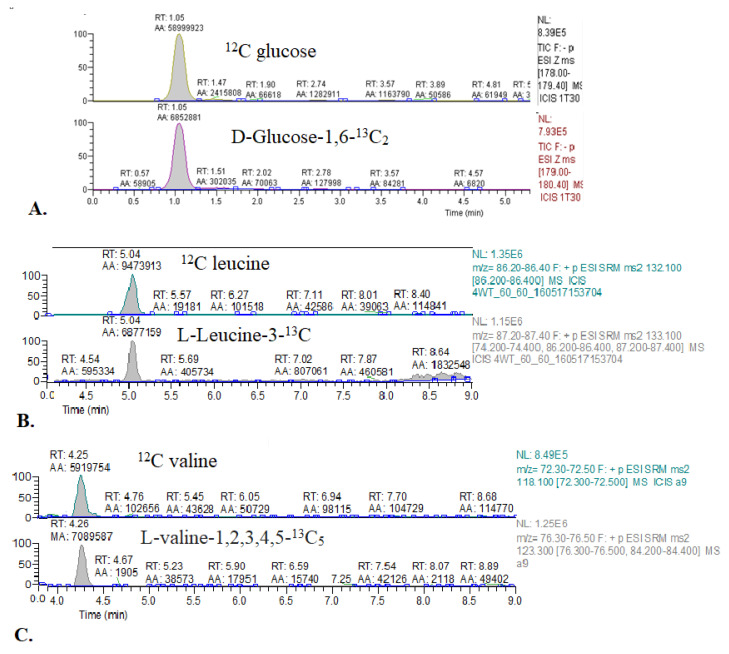
Representative chromatograms of ^13^C glucose and ^13^C BCAA enrichment in mouse blood. (**A**) ^12^C glucose and glucose-1,6-^13^C_2_. (**B**) ^12^C leucine and leucine-3-^13^C. (**C**) ^12^C valine and valine-1,2,3,4,5-^13^C_5_.

The ^13^C glucose enrichment in blood was measured using liquid chromatography-mass spectrometry—an LCQ-Deca XP mass detector (Thermo Finnigan, San Jose, CA, USA). The chromatography column (Agilent Technologies, Santa Clara, CA, USA) (Zorbax NH2, 50 mm × 2.1 mm) temperature was 25 °C. The mobile phases consisted of the following buffers: (A) 5 mM ammonium acetate and 5 mM ammonium hydroxide, and (B) 100% acetonitrile. Firstly, for chromatography system equilibration, the column was eluted with a phase composed of 10% buffer A and 90% B. After 4 min, the phase composition was 40% A, 60% B. These conditions were held for 5 min, and then the gradient elution return to initial conditions. The mobile phase flow rate was 300 µL/min and the injection volume was 2 µL. Fragments containing ^12^C and ^13^C glucose were detected in negative electrospray ionization with the selected ion monitoring (SIM) mode—for ^12^C glucose *m/z* 178.00–179.40 and *m/z* 179.00–180.40 for d-glucose-1,6-^13^C_2_ (Figure A1A).

**Figure A2:**
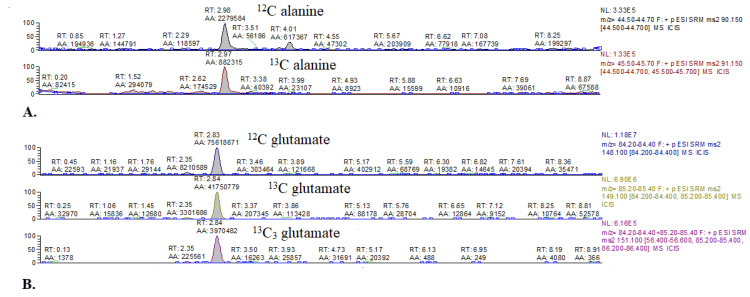
Representative chromatograms of ^13^C alanine and ^13^C glutamate enrichment in mouse hearts. (**A**) ^12^C alanine and 3-^13^C alanine. (**B**) ^12^C glutamate, 4-^13^C-glutamate, and 1,2,3-^13^C_3_ glutamate.

The heart (analysis of ^13^C alanine and ^13^C glutamate enrichment) and blood (analysis of ^13^C leucine and ^13^C valine enrichment) extracts were analyzed by liquid chromatography–mass spectrometry using a TSQ-Vantage triple quadrupole mass detector (Thermo Fisher, Waltham, MA, USA) linked to the Surveyor chromatography system (Thermo Fisher, Waltham, MA, USA). The temperature of the chromatography column (Phenomenex Synergi Hydro RP 5 mm × 2 mm) was 25 °C. The mobile phases consisted of (A) 5 mM of nonafluoropentanoic acid (NFPA) and (B) 0.1% formic acid in acetonitrile. Initially, for chromatography system equilibration, the column was eluted with a phase composed of 90% buffer A and 10% B. After two minutes, the phase composition was 70% A, 30% B. Those chromatography conditions were held for 4.5 min and then the gradient elution comprised only 100% of buffer B. The mobile phase flow rate was 200 µL/min and the injection volume was 2 µL. Mass detection was carried out in a positive heated electrospray ionization with fragmentation mode (Tandem MS). In the blood extracts, fragments containing C^13^ in the leucine or C^13^ in the valine structure were monitored (Figure A1B,C). ^12^C leucine was monitored at 132.10 *m/z* for the primary ion and *m/z* 86.20–86.40 for the derivative. l-leucine-3-^13^C was monitored at 133.10 *m/z* for the primary ion and *m/z* 87.20–87.40 for the derivative. ^12^C valine was monitored at 118.10 *m/z* for the primary ion and *m/z* 72.30–72.50 for the derivative. L-valine-1,2,3,4,5-^13^C_5_ was monitored at 123.30 *m/z* for the primary ion and *m/z* 76.30–76.50 for the derivative. In the heart extracts, fragments containing C^13^ in alanine or C^13^ in glutamate structure were monitored (Figure A2A,B). ^12^C glutamate was monitored at *m/z* 148.10 for the primary ion and *m/z* 84.20–84.40 for the derivative. In the case of 4-^13^C glutamate *m/z* for the primary ion, 149.10 and *m/z* 84.20–84.40, 85.20–85.40 for the derivative ion, and 151.10 *m/z* for the primary and 85.30 *m/z* for the derivative ion for 1,2,3-^13^C_3_ glutamate were monitored. ^12^C alanine was monitored at 90.15 *m/z* for the primary ion and *m/z* 44.50–44.70 for the derivative, whereas 3-^13^C alanine was monitored at 91.15 *m/z* for the primary ion and *m/z* 44.50–44.70 for the derivative (Table A1).

### 4.5. Statistical Analyses

Statistical significance was evaluated using Student’s t-test. A value of *p* < 0.05 was used to denote statistical significance, and results are expressed as mean ± SEM. All statistics were carried out using GraphPad Prism 5.00 (GraphPad Software, San Diego, CA, USA).

## 5. Conclusions

It is crucial to note that the methods created are focused on comparative analysis and do not determine exact rates or detect all metabolites that arise from ^13^C glucose and ^13^C BCAA. Furthermore, the analyses of changes in ^13^C-labeled metabolites do not take into account the potential utilization of glucose and BCAAs from endogenous stores. Finally, due to systemic isotopomer administration, other organs such liver, brain, or skeletal muscle may convert labeled substrates before the ^13^C reaches the heart. However, the blood isotopomer enrichment of blood BCAA or glucose pools was very substantial, exceeding 40%, and time was too short for secondary labeling. Besides, response to established strategies that modulate cardiac substrate preference was consistent with their mechanism of action. Furthermore, stable enrichment of ^13^C glutamate in the heart after ^13^C glucose and BCAA administration as well as distinct differences in ^13^C enrichment of metabolites in heart tissue vs. blood suggest the suitability of our protocols to investigate cardiac-driven changes in glucose and branched-chain amino acid metabolism. In future work, the quantification of additional ^13^C-labeled metabolites that are specific to the cardiac compartment (e.g., organic acids) as well as investigation of other ^13^C-labeled substrates, e.g., fatty acids, may demonstrate a more comprehensive picture of cardiac substrate preference and metabolism.

Besides these limitations, our methods allow for stable isotopomer enrichment that is sensitive to pharmacotherapy and substrate usage changes under pathological conditions. Importantly, it is simpler, faster, and easier to adapt in most laboratories in contrast to more sophisticated and accurate methods, such as NMR analysis used in cardiac metabolism research [50]. In summary, our methods allowed for fast and simple estimation of cardiac glucose and BCAA (leucine or valine) use in mice and have the potential to analyze changes in pathology in mouse animal models and after pharmacological interventions.

## Data Availability

The authors declare that the data supporting the findings of the study are available within the article.

## Appendix A

**Table A1:** Summary of compounds monitored by LC/MS with primary and derivative ion values [*m/z*].

Compound	Primary Ion (*m/z*)	Derivative Ions (*m/z*)
Glucose	178.00–179.40	-(SIM mode)
1,6-^13^C_2_ glucose	179.00–180.40	-(SIM mode)
Leucine	132.10	86.20–86.40
3-^13^C leucine	133.10	87.20–87.40
Valine	118.10	72.30–72.50
1,2,3,4,5-^13^C_5_ valine	123.30	76.30–76.50
Glutamate	148.10	84.20–84.40
4-^13^C glutamate	149.10	84.20–84.40, 85.20–85.40
1,2,3-^13^C_3_ glutamate	151.10	85.30
Alanine	90.15	44.50–44.70
3-^13^C alanine	91.15	44.50–44.70
